# Supplementary material for: Early evolution of the ecdysozoan body plan
Source: eLife. 2024 Jul 8;13:RP94709. doi: 10.7554/eLife.94709 (PMC11231812; doi:10.7554/eLife.94709)
Supplement: Figure 3—source data 1. [file elife-94709-fig3-data1.docx]

**Morphological characters used in the present phylogenetic analyses.**

Characters from those three studies are marked as such with a dagger (†) for Ou et al.[S1] and an asterisk for those from Vinther & Parry[S2] (*). New characters are indicated in bold.

1. †Collar complex
   1. absent
   2. present
2. †Multicellularity with extracellular matrix 0 absent

1 present

1. †Septate junctions (SJs) 0 absent

1 present

1. †Tight junctions (TJs) 0 absent

1 present

1. †Gap junctions (GJs) 0 absent

1 present

1. †Adherens junctions (AJs) 0 absent

1 present

1. †Hemidesmosomes
2. absent
3. present
4. †Epithelia
5. absent
6. present
7. †Basal laminae
8. absent
9. present
10. †Collagen
11. absent
12. present
13. †Nerve cells
14. absent
15. present
16. †Acetylcholine used as a neurotransmitter 0 absent

1 present

1. †Diffuse nervous system 0 absent

1 present

1. †Epidermis with pulsatite bodies 0 absent

1 present

1. *Ciliated epidermis.

0 absent

1 present

1. *Multiciliate epidermal cells 0 absent

1 present

1. *Ciliation restricted anteriorly. 0 absent

1 present

1. *Ciliated corona with paired nerves. 0 absent

1 present

1. *Ventral locomotory cilia. 0 absent

1 present

1. *Ventral surface modified into foot. 0 absent

1 present

1. †Xenacoelomorph cilia 0 absent

1 present

1. †Striated ciliary rootlets
2. absent
3. present
4. †Diploblasts built of two germ layers 0 absent

1 present

1. †Triploblasts built of three germ layers 0 absent

1 present

1. †Spiral cleavage
2. absent
3. present
4. *Apical cross.
5. absent
6. present
7. †4d mesentoblast.
8. absent
9. present
10. †Colloblasts
11. absent
12. present
13. †Coelenteron (gastrovascular cavity) 0 absent

1 present

1. †Cnidae
2. absent
3. present
4. †Structure of mitochondrial DNA 0 circular

1 linear

1. †Actinopharynx
2. absent
3. present
4. †Siphonoglyph (sulcus)
5. †Planulae
6. absent
7. present
8. †Polyp stage
9. absent
10. present
11. †Medusoid stage
12. absent
13. present
14. †Through-gut
15. absent
16. present

Note: The presence of a mouth on the ventral side of *Beretella* is uncertain and coded with a question mark. No other opening such as a possible anus occurs in *Beretella*. *Saccorhytus* has no known gut features [S3, S4] and is coded “0”.

1. †U-shaped gut
2. absent
3. present
4. *Dorsal anus or anal pore. 0 absent

1 present

1. *Ventral mouth.
2. absent
3. present

Note: See main text and note in Character 37.

1. Adult terminal mouth.
2. absent
3. present

Note: Modified from Vinther and Parry [S2] to clarify that this character describes the anterior terminal mouth in the adult form[S5-S7].

1. †Body divided with distinct inflated head and neck region. 0 absent

1 present

Note: Neither *Saccorhytus* nor *Beretella* have a differentiation of the body (e.g. distinct head and neck).

1. †Bipartite gut with cuticular pharynx.

0 absent

1 present

Note: see notes in Character 42.

1. †Nephridia

0 absent

1 present

1. *Fate of blastopore.
2. protostomy
3. deuterostomy
4. amphistomy
5. unique

46. Annelid type cuticle

0 absent

1 present

47. †Body cuticle with chitin. 0 absent

1 present

48. †Body cuticle with alpha-chitin 0 absent

1 present

49. † Body cuticle molted

0 absent

1 present

50. †Lorica in larvae.

- 1. absent

1present

51. Lobopods or segmented limbs

0 absent

1 present

52. †Limb type

1. lobopods
2. arthrodized limbs

53. *Anteriorly facing last pair of limbs. 0 absent

1 present

54. †Slime papillae

1. absent
2. present

55. †Telescoping mouth cone with protrudable stylets

0 absent

1 present

56. †Respiration via metameric tracheae and spiracles

0 absent

1 present

Note: Neither *Saccorhytus* nor *Beretella* show such structure, they are scored “0”.

57. †Mixocoel (haemocoel) surrounded by segmented mesoderm 0 absent

1 present

58. †Teloblastic segmentation 0 absent

1 present

Note: Neither *Saccorhytus* nor *Beretella* show such structure, they are scored “0”.

59. †Longitudinal ventral nerve cord(s) 0 absent

1 present

60. *Paired VNC. (Sipunculan form) 0 absent

1 present

61. *Circumoral nerve ring. 0 absent

1 present

62. *Stomatogastric nerve plexi. 0 absent

1 present

63. Major ventral nerve plexus in trunk. 0 absent

1 present

64. Circum-pharyngeal, collar-shaped brain with anterior and posterior rings of perikarya separated by a ring-shaped neuropil.

1. absent
2. present

65. †Introvert with scalid rings 0 absent

1 present

Note: No introvert seems to be present in *Acosmia*, *Saccorhytus* and *Beretella* are both sac-like and devoid of introvert. They are coded “0”.

66. †Flosculi

0 absent

1 present

Note: Neither *Saccorhytus* nor *Beretella* show such structure. they are scored “0”.

67. Immunoreactivity of horseradish peroxidase (HRP)

0 absent

1 present

68. †Trochophores

1. absent
2. present

Note: Neither *Saccorhytus* nor *Beretella* show such structure, they are scored “0”.

69. *Prototroch.

1. absent
2. present

Note: This character is coded as “-” if character 68 is absent.

70. *Apical organ.

1. absent
2. present

Note: Neither *Saccorhytus* nor *Beretella* show such structure, they are scored “0”.

71. *Apical organ with muscles extending to the hyposphere. 0 absent

1 present

72. †Segmental metanephridia sacculus 0 absent

1 present

73. †Chaetae.

1. absent
2. present

74. †Chaetae in bundles. 0 absent

1 present

75. †Mineralised chaetae. 0 absent

1 present

76. *Serially repeated chaetal bundles. 0 absent

1 present

77. †Parapodia with dorsal and ventral branches 0 absent

1 present

78. †Radula

1. absent
2. present

Note: No evidence for a radula in *Beretella*, it is coded with a question mark.

79. *Radula tooth rows.

1. few rows
2. many rows

80. *Chitinous pharyngeal structure. 0 absent

1 present

Note: No evidence for a pharyngeal structure in *Beretella*, it is coded “0”.

81. *Tube like support rods in jaw. 0 absent

1 present

82. *Grasping spines.

1. absent
2. present

83. *Teeth (as in Chaetognatha) 0 absent

1 present

84. *Anterior teeth (as in Chaetognatha). 0 absent

1 present

85. *Posterior teeth (as in Chaetognatha). 0 absent

1 present

86. *Jaw apparatus inside mastax. 0 absent

1 present

87. *Jaw apparatus forming a ventral membrane 0 absent

1 present

88. *Jaw forming clusters of homonomous elements. 0 absent

1 present

89. *Ctenidia.

1. absent
2. present

90. *Mantle cavity.

1. absent
2. present

91. †Eversible proboscis surrounded by rhynchocoel 0 absent

1 present

92. *Subterminal anus. 0 absent

1 present

93. *Tripartite body plan with septum. 0 absent

1 present

94. Posterior lateral trunk fins. 0 absent

1 present

95. Anterior lateral fins. 0 absent

1 present

96. *Caudal fin.

1. absent
2. present

97. *Fins supported by rays. 0 absent

1 present

98. *Phragms.

1. absent
2. present

99. *Phragms in trunk.

1. absent
2. present

100. *Phragms in tail.

1. absent
2. present

101. *Stomatogastric nerve plexi.

0 absent

1 present

102. Major nerve plexus in trunk.

0 absent

1 present

103. *Lateral sensory antennae. 0 absent

1 present

104. *Posterior adhesion structure. 0 absent

1 present

105. *Head tentacles.

1. absent
2. present

106. *Head with hood.

1. absent
2. present

107. *AP axis.

1. absent
2. present

Note: The low elevated end of *Beretella* is tentatively interpreted as the anterior one, based on the orientation of the spiny sclerites (see text).

108. *Dorsoventral axis 0 absent

1 present

Note: The flattened side of *Beretella* is interpreted as the ventral one, based on the opposite convex side armed with string spines (the assumed dorsal side; see text).

109. †Origin of mesoderm

0 from the blastopore lips and as ectomesoderm 1 from the walls of the archenteron or neural crest

110. †Radial cleavage

1. absent
2. present

111. †Coelom

1. absent
2. present

112. †Coelom formation

1. schizocoely
2. enterocoely

113. *Serially repeated paired coelomic cavities. 0 absent

1 present

114. †Trimeric coelom

1. absent
2. present

115. †Pharyngeal slits

1. absent
2. present

116. †Endostyle (or homologues) 0 absent

1 present

117. †Notochord

1. absent
2. present

118. †Stomochord

1. absent
2. present

119. †Haemal system with axial complex 0 absent

1 present

120. †Calcareous endoskeleton composed of separate ossicles 0 absent

1 present

121. †Tornaria type larva 0 absent

1 present

122. †Longitudinal dorsal nerve cord 0 absent

1 present

123. †Zig zag myomeres

0 absent

1 present

124. Endothelium that lines the inner wall of blood vessels

0 absent

1 present

125. †Neural crest

1. absent
2. present

126. †Neurogenic placodes 0 absent

1 present

127. †Body symmetry 0 radial

1. bilateral
2. biradial

Note: Its pharyngeal elements seem to be radially arranged elements. The body symmetry of *Acosmia* is therefore coded “0”

128. †Mesoglea

1. absent
2. present

129. †Cydippid larvae

1. absent
2. present

130. †Ciliary rosettes

1. absent
2. present

131. †Lophophore

1. absent
2. present

132. †Hox/ParaHox genes 0 absent

1 present

133. *ftz.

1. absent
2. present

134. *Ubx/abd-A.

0 absent

1 present

135. *AbdB.

1. absent
2. present

136. *lox5.

1. absent
2. present

137. *Duplication of UbdA into lox4 and lox2. 0 absent

1 present

138. *Post2.

1. absent
2. present

139. *Duplication into Post1 and Post2. 0 absent

1 present

140.*MedPost.

1. absent
2. present

141. Annulated cuticle. Character 37 in Smith & Caron [S8]. 0 absent

1 present

Note: Neither *Saccorhytus* nor *Beretella* show such cuticular structure; they are coded “0”.

142. Radial pharyngeal armature.

1. absent
2. present

Note: Modified from character 13 in Smith & Caron [S8] (sclerotized pharyngeal ‘teeth’) to include all ecdysozoan radial pharyngeal armature (including kinorhynch pharyngeal styles, loriciferan placoids etc.). *Saccorhytus* has no pharyngeal armature and is therefore coded “0”. Incomplete ventral side of *Beretella* shows uncertain of pharyngeal structures and is tentatively coded “0”. Pharyngeal elements of *Acosmia* seem to be radially arranged (see note in Character 125) and is coded “1”.

143.Posteriorly directed mouth opening. Modified from character 7 in Smith & Caron[S8]. 0 absent

1 present

144. Serially repeated mid-gut glands. Character 53 in Smith & Caron[S8].

0 absent

1 present

145. Lobopodian-style ocelli.

1. absent
2. present

Note: This character describes the paired visual organs present in taxa such as *Hallucigenia sparsa* and *Luolishania longicruris* – see characters 16 – 18 in Smith & Caron [S8].

146.Compound eyes.

1. absent
2. present

Note: Characters 16 – 18 in Smith & Caron [S8].

147. Cephalic sensillae.

1. absent
2. present

Note: The anterior mechano-sensroy and chemo-sensory organs of nematodes form rings with a 6 + 6 + 4 pattern around the anterior region [S9].

148. Placids.

1. absent
2. present

Note: Placids are anterior plates that enclose the retracted introvert in kinorhynchs [S10].

149. Elongate spines at the base of Zone II (collar).

1. absent
2. present

Inapplicable if taxon lacks scalid covered introvert. Character 13 in Wills et al. [S11].

150. Introvert scalids arranged into parallel longitudinal rows.

1. absent
2. present

Inapplicable if taxon lacks scalid covered introvert. Modified from character 9 in Wills et al. [S11].

151. Integument sclerotized and connected by arthrodial membranes. Character 35 in Smith & Caron[S8].

1. absent
2. present

152. Tergites associated with arthrodial membranes. Extension of Character 35 in Smith & Caron[S8].

1. absent
2. present

153. Sternites associated with arthrodial membranes. Character 36 in Smith & Caron [S8].

0 absent

1 present

154. Cephalic shield. Character 3 in Smith & Caron[S8].

0 absent

1 present

155. Musculature exclusively longitudinal.

1. absent
2. present

Note: Nematoid bodyplans comprise only longitudinal muscles [S12].

156. Skeletal musculature metamerically arranged. Character 9 in Smith & Caron[S8]. 0 absent

1 present

157. Jointed, reflexed introvert scalids.

1. absent
2. present

Inapplicable if taxon lacks scalid covered introvert.

Note: This character refers to the elongate anterior spinoscalids of loriciferans, which show a clear elbow-like joint [S13].

158. Eversible pharynx. Character 12 in Smith & Caron[S8]. 0 absent

1 present

159. Paired metameric epidermal specialisations. Character 41 in Smith & Caron[S8]. 0 absent

1 present

160. Metameric epidermal specialisations: spinose (as in hallucigeniids and luolishaniids) 0 absent

1 present

161. Metameric epidermal specialisations: net-like (as in *Microdictyon*) 0 absent

1 present

162. Metameric epidermal specialisations: saddle-like (as in *Cardiodictyon*) 0 absent

1 present

163. Metameric epidermal specialisations: node-like (as in *Onychodictyon*) 0 absent

1 present

164. Terminal claws on lobopods. Character 63 in Smith & Caron[S8].

1. absent
2. present

Inapplicable if taxon lacks paired appendages

165. Sclerites/claws consist of a stack of constituent elements (cone in cone structure). Character 48 in Smith & Caron[S8].

1. absent
2. present

Inapplicable if taxon lacks claws, sclerites etc.

166. Uniform appendages.

1. absent
2. present

Inapplicable if taxon lacks paired appendages.

Note: This character describes the state of having metameric paired appendages lacking any differentiation (i.e. an absence of tagmosis). For example, the Chengjiang lobopodians such as *Paucipodia inermis, Diania cactiformis* and *Microdictyon sinicum* display a uniform series of appendages along their body.

167. Pre-ocular appendage pair structurally differentiated from trunk appendages. Character 20 in Smith & Caron ^8^.

1. absent
2. present

Inapplicable if taxon lacks paired appendages

168. Pre-ocular appendage pair antenniform.

1. absent
2. present

Inapplicable if taxon lacks paired appendages.

Note: This character describes the antenniform anteriormost appendages (possibly protocerebral) of some onychophorans and some arthropods.

169. Spines/spinules on pre-ocular (protocerebral) appendage. Character 30 in Smith & Caron[S8]. 0 absent

1 present

Inapplicable if taxon lacks paired appendages

170. Sclerotization of pre-ocular (protocerebral) limb pair. Character 21 in Smith & Caron[S8]. 0 absent

1 present

Inapplicable if taxon lacks paired appendages

171. Pre-ocular (protocerebral) limb pair with arthrodial membranes. Character 22 in Smith & Caron[S8].

1. absent
2. present

Inapplicable if taxon lacks paired appendages

172. Pre-ocular (protocerebral) appendages mechanically fused. Character 28 in Smith & Caron[S8]. 0 absent

1 present

Inapplicable if taxon lacks paired appendages

173. Deutocerebral limb pair structurally differentiated from trunk appendages. Character 24 in Smith & Caron [S8].

1. absent
2. present

Inapplicable if taxon lacks paired appendages

174. Sclerotized post-ocular (post-protocerebral) body appendages with arthrodial membranes. Character 19 in Smith & Caron[S8].

1. absent
2. present

Inapplicable if taxon lacks paired appendages

175. Laterally expanded swimming flap as inner branch of lobopod. Character 23 in Smith & Caron[S8].

1. absent
2. present

Inapplicable if taxon lacks paired appendages

176. Biramy (exite and endopod fused). Character 57 in Smith & Caron[S8]. 0 absent

1 present

Inapplicable if taxon lacks paired appendages

177. Anterior-posterior tagmosis of non-arthropodized appendages.

1. absent
2. present

Inapplicable if taxon lacks paired appendages.

Note: Some lobopodians have a uniform set of appendages (e.g. *Paucipodia inermis*), or a single specialised anterior appendage (e.g. *Aysheaia pedunculata*). Others display tagmosis represented by multiple specialised anterior appendages – resulting in an anterior-posterior tagmosis (e.g. *Hallucigenia sparsa*, *Cardiodictyon catenulum*, Luolishaniidae and Onychophora).

178. Elongated anterior lobopodous limbs, differentiated from shorter posterior two short limbs. Character 72 in Yang et al. [S14].

1. absent
2. present

Inapplicable if taxon lacks paired appendages

179. Setae on anterior specialized lobopods. Character 60 in Smith & Caron[S8]. 0 absent

1 present

Inapplicable if taxon lacks paired appendages

180. Caudal appendage. Character 45 in Wills et al. [S11]. 0 absent

1 present

181. Two rows of ventral trunk papillae. Character 30 in Wills et al. [S11]. 0 absent

1 present

182. Posterior hooks in a ring. Character 30 in Wills et al. [S11]. 0 absent

1 present

183. Posterior pair of hooks. Character 30 in Wills et al. [S11]. 0 absent

1 present

184. Swollen, hook-bearing posterior.

1. absent
2. present

Note: This character describes the anchoring swollen feature of *Facivermis yunnanicus* [S15].

185. Radially arranged circumoral structures. Character 9 in Smith & Caron[S8].

0 absent

1 present

Note: *Saccorhytus* has such characteristics (coded ”1”). A single specimen of *Beretella* show a tiny spine in the central area of its ventral side that is supposed to have accommodated the mouth opening. It is tentatively interpreted as the remain of a possible oral spiny apparatus (as in *Saccorhytus*), but it is better coded "?". *Acosmia* bears a circlet of circumoral elements (i.e. lip in [S15]).

**186. Paired, cuticular sclerite and non-serial arrangement on the dorsal body**

0 absent

1 present

Note: Non-serial arrangement of sclerites is relative to serial arrangement that sclerites along the trunk are paired in the annulations and align in longitudinal row (e.g. *Microdictyon*, *Cricocosmia*). Non-serial arrangement refers to the presence of paired sclerites but not in annulations and longitudinal row (e.g. *Saccorhytus*, *Beretella*). This feature is present in both *Saccorhytus* and *Beretella* but lacks in other sclerite-bearing ecdysozoans.

187. Cuticle surface with ornament of tessellating plates. Modified from character 91 from Harvey et al. [S16].

0 absent

1 present

188. Spinose sclerites with radial folds. Character 39 from Han et al. [S3].

0 absent

1 present

Note: These folds are present in *Saccorhytus* (coded ”1”) not in *Beretella* (coded as “0”).

**189.Reticulate polygons on the cuticle may correspond to underlying pidermal cells.**

0 absent

1 present

Note: This character is present in all extant ecdysozoans [S17] and *Beretella* but absent in *Saccorhytus* (chevron pattern) [S3].

**190. Number of paired large dorsal sclerites (non-serial arrangement):**

0 two

1 four

Note: This is related to character 184. Inapplicable if character 184 is absent. *Saccorhytus* seems to have four pairs of such sclerites [S3, S4]. *Beretella* only has two.

**191. Bilaterally symmetrical arrangement dorsal small spines**

0 absent

1 present

Note: The distribution of these small spines is more irregular in *Saccorhytus* [S3] than in *Beretella*.

**192. Orientation of tips of spinose sclerites:**

0 perpendicular to the body surface

1 posteriorly directed

**193. Body differentiation with definitive head and trunk at least in certain stages of life cycle:**

0 absent

1 present

Note: It is absent in saccorhytids. Body differentiation remains questionable in *Acosmia* but is present in the vast majority of bilaterians.

**Supplementary references**

S1. Ou, Q., Han, J., Zhang, Z., Shu, D., Sun, G., and Mayer, G. (2017). Three Cambrian fossils assembled into an extinct body plan of cnidarian affinity. Proceedings of the National Academy of Science *114*, 8835-8840.

S2. Vinther, J., and Parry, L.A. (2019). Bilateral jaw elements in *Amiskwia sagittiformis* bridge the morphological gap between gnathiferans and chaetognaths. Current Biology *29*, 881-888 e881.

S3. Han, J., Conway Morris, S., Ou, Q., Shu, D.G., and Huang, H. (2017). Meiofaunal deuterostomes from the basal Cambrian of Shaanxi (China). Nature *542*, 228-231.

S4. Liu, Y., Carlisle, E., Zhang, H., Yang, B., Steiner, M., Shao, T., Duan, B., Marone, F., Xiao, S., and Donoghue, P.C.J. (2022). *Saccorhytus* is an early ecdysozoan and not the earliest deuterostome. Nature *609*, 541-546.

S5. Ortega-Hernandez, J., Janssen, R., and Budd, G.E. (2017). Origin and evolution of the panarthropod head - A palaeobiological and developmental perspective. Arthropod Struct Dev *46*, 354-379.

S6. Ortega-Hernandez, J., Janssen, R., and Budd, G.E. (2019). The last common ancestor of Ecdysozoa had an adult terminal mouth. Arthropod Structure & Development *49*, 155-158.

S7. Nielsen, C. (2019). Was the ancestral panarthropod mouth ventral or terminal? Arthropod Structure & Development *49*, 152-154.

S8. Smith, M.R., and Caron, J.B. (2015). *Hallucigenia*'s head and the pharyngeal armature of early ecdysozoans. Nature *523*, 75-78.

S9. Schmidt-Rhaesa, A. (2014). Handbook of Zoology, Volume 2, (Germany: De Gruyter).

S10. Neuhaus, B., and Sørensen, M.V. (2013). Populations of Campyloderes sp. (Kinorhyncha, Cyclorhagida): One global species with significant morphological variation? Zoologischer Anzeiger *252*, 48-75.

S11. Wills, M.A., Gerber, S., Ruta, M., and Hughes, M. (2012). The disparity of priapulid, archaeopriapulid and palaeoscolecid worms in the light of new data. Journal of Evolutionary Biology *25*, 2056-2076.

S12. Schmidt-Rhaesa, A. (2013). Nematomorpha. In Gastrotricha, Cycloneuralia and Gnathifera, Volume 1, A. Schmidt-Rhaesa, ed. (Germany: De Gruyter), pp. 29-146.

S13. Bang-Berthelsen, I.H., Schmidt-Rhaease, A., and Møbjerg, K. (2013). Loricifera. In Handbook of zoology-Gastrotricha, Cycloneuralia and Gnathifera, Volume 1, A. Schmidt-Rhaesa, ed. (Germany: De Gruyter), pp. 349-370.

S14. Yang, J., Ortega-Hernández, J., Gerber, S., Butterfield, N.J., Hou, J.-b., Lan, T., and Zhang, X.-g. (2015). A superarmored lobopodian from the Cambrian of China and early disparity in the evolution of Onychophora. Proceedings of the Academy of Natural Sciences of the United States of America *112*, 8678-8683.

S15. Howard, R.J., Hou, X., Edgecombe, G.D., Salge, T., Shi, X., and Ma, X. (2020). A tube-dwelling early Cambrian lobopodian. Current Biology *30*, 1-8.

S16. Harvey, T.H., Dong, X., and Donoghue, P.C. (2010). Are palaeoscolecids ancestral ecdysozoans? Evol Dev *12*, 177-200.

S17. Wang, D., Vannier, J., Yang, X.G., Sun, J., Sun, Y.F., Hao, W.J., Tang, Q.Q., Liu, P., and Han, J. (2020). Cuticular reticulation replicates the pattern of epidermal cells in lowermost Cambrian scalidophoran worms. Proceedings of the Royal Society B *287*, 20200470.
