# Supplementary material for: Early evolution of the ecdysozoan body plan
Source: eLife. 2024 Jul 8;13:RP94709. doi: 10.7554/eLife.94709 (PMC11231812; doi:10.7554/eLife.94709)
Supplement: Supplementary file 1. — (a) Measurements of Beretella. L, length; W, width; H, height; ae, anterior end; B, body; pe, posterior end; PP, polygonal net-like pattern; tp, tiny spine; VO, ventral opening;?, no accurate measurement possible. (b) Length/width ratio of Beretella and Saccorhytus. (c) Similarities and differences between Beretella and Saccorhytus. AP, antero-posterior; DV, dorso-ventral side; LR, left-right. (d) Ancestral character state reconstructions for the topology where Cycloneuralia is monophyly. Values of ancestral character state reconstructions. 0=absence of character, 1=presence of character, P=posterior probability. TGE, total-group Ecdysozoa; CGE, crown-group Ecdysozoa, SA, Saccorhytida. (e) Ancestral character state reconstructions for the topology where Cycloneuralia is paraphyletic. Values of ancestral character state reconstructions. 0=absence of character, 1=presence of character, P=posterior probability. TGE, total-group Ecdysozoa; CGE, crown-group Ecdysozoa, SA, Saccorhytida. [file elife-94709-supp1.docx]

**Supplementary File 1a. Measurements of *Beretella*.** L, length; W, width; H, height; ae, anterior end; B, body; pe, posterior end; PP, polygonal net-like pattern; tp, tiny spine; VO, ventral opening; ?, no accurate measurement possible.

| specimen no. | | | CUBar138-12 | | | CUBar75-45 | | | CUBar171-5 | | | CUBar121-8 | | | CUBar99-19 | | | CUBar136-9 | | | CUBar136-11 | | |
| --- | --- | --- | --- | --- | --- | --- | --- | --- | --- | --- | --- | --- | --- | --- | --- | --- | --- | --- | --- | --- | --- | --- | --- |
|  |  |  | L (in μm) | W (in μm) | H (in μm) | L (in μm) | W (in μm) | H (in μm) | L (in μm) | W (in μm) | H (in μm) | L (in μm) | W (in μm) | H (in μm) | L (in μm) | W (in μm) | H (in μm) | L (in μm) | W (in μm) | H (in μm) | L (in μm) | W (in μm) | H (in μm) |
| B |  | | 1960 | 1225 | 1097 | 1170 | 975 | ? | 1470 | 1159 | ? | 2616 | 1972 | 1218 | ? | ? | ? | 1411 | 1296 | ? | 2900 | 2450 | ? |
| VO |  | | 665 | 584 | ? | ? | ? | ? | ? | ? | ? | 1175 | 1607 | ? | ? | ? | ? | ? | ? | ? | ? | ? | ? |
|  | S1 | 1 | 110 | 110 | ? | ? | ? | ? | 97 | 97 | ? | ? | ? | ? | ? | ? | ? | ? | ? | ? | ? | ? | ? |
|  |  | 2 | 140 | 140 | ? | ? | ? | ? | 126 | 126 | ? | ? | ? | ? | ? | ? | ? | ? | ? | ? | ? | ? | ? |
|  |  | 3 | 275 | 180 | ? | 181 | 181 | ? | 165 | 165 | ? | ? | ? | ? | 238 | 190 | ? | ? | ? | ? | ? | ? | ? |
|  |  | 4 | 187 | 187 | ? | ? | ? | ? | 372 | 214 | ? | ? | ? | ? | 318 | 157 | ? | 260 | 106 | ? | ? | ? | ? |
|  | L/RS2 | 1 | 87~91 | 54 | ? | ? | ? | ? | 124 | 124 | ? | ? | ? | ? | ? | ? | ? | ? | ? | ? | ? | ? | ? |
|  |  | 2 | 177~216 | 78~150 | ? | ? | ? | ? | 174 | 174 | ? | ? | ? | ? | 123 | 123 | ? | ? | ? | ? | ? | ? | ? |
|  |  | 3 | 200~224 | 107~133 | ? | ? | ? | ? | 179 | 179 | ? | ? | ? | ? | 203 | 203 | ? | 103~115 | 71 | ? | ? | ? | ? |
|  |  | 4 | 102~113 | 68~72 | ? | ? | ? | ? | 175 | 175 | ? | ? | ? | ? | 193 | 193 | ? | 92~99 | 92 | ? | ? | ? | ? |
|  |  | 5 | 106~137 | 80~110 | ? | ? | ? | ? | 168 | 168 | ? | 109 | 109 | ? | 193~258 | 193~258 | ? | 124~148 | 93~106 | ? | ? | ? | ? |
|  | S3-1 |  | 593 | 323 | ? | 214 | 214 | ? | ? | ? | ? | 304 | ? | ? | ? | ? | ? | 404 | ? | ? | 357 | ? | ? |
|  | L/RS3 | 2 | 700~727 | 422 | ? | 495~500 | 350~392 | ? | 485 | ? | ? | 440 | ? | ? | ? | ? | ? | 330 | 304 | ? | 524 | 384 | ? |
|  |  | 3 | 749 | 455 | ? | 507 | ? | ? | 587 | 323 | ? | 451 | 287 | ? | 773 | ? | ? | 446 | ? | ? | 533 | 411 | ? |
|  | S3-4 |  | 340 | 340 | ? | ? | ? |  | ? | ? | ? | 201 | ? | ? | 342 | 315 | ? | 337 | ? | ? | ? | ? | ? |
|  | L/RS4 | 1 | 191 | 123 | ? | ? | ? | ? | ? | ? | ? | 138 | ? | ? | ? | ? | ? | 151 | 151 | ? | 173 | ? | ? |
|  |  | 2 | 137 | 121 | ? | ? | ? | ? | 196 | 196 | ? | 166 | ? | ? | ? | ? | ? | ? | ? | ? | ? | ? | ? |
|  |  | 3 | 149 | 105 | ? | 83 | 83 | ? | 221 | 221 | ? | ? | ? | ? | ? | ? | ? | ? | ? | ? | 186 | ? | ? |
|  |  | 4 | 160 | 137 | ? | 110 | 110 | ? | 196 | 196 | ? | 166 | ? | ? | ? | ? | ? | 195 | ? | ? | 191 | ? | ? |
|  |  | 5 | 191 | 170 | ? | 115 | 115 | ? | 216 | 216 | ? | ? | ? | ? | ? | ? | ? | ? | ? | ? | ? | ? | ? |
|  |  | 6 | 162 | 138 | ? | 141 | 141 | ? | 236 | 236 | ? | 124~ 164 | ? | ? | 316~332 | ? | ? | ? | ? | ? | ? | ? | ? |
|  | S5 | 1 | 78 | 78 | ? | ? | ? | ? | 101~ 141 | 98~122 | ? | 113 | 113 | ? | ? | ? | ? | ? | ? | ? | ? | ? | ? |
|  |  | 2 | 68 | 68 | ? | ? | ? | ? | ? | ? | ? | 105 | 105 | ? | ? | ? | ? | ? | ? | ? | ? | ? | ? |
|  |  | 3 | 110 | 110 | ? | 95 | 95 | ? | ? | ? | ? | ? | ? | ? | ? | ? | ? | ? | ? | ? | 107 | ? | ? |
|  |  | 4 | 82 | 82 | ? | ? | ? | ? | 146 | 146 | ? | ? | ? | ? | ? | ? | ? | ? | ? | ? | ? | ? | ? |
|  |  | 5 | 74 | 74 | ? | 92 | 92 | ? | ? | ? | ? | 121 | 121 | ? | ? | ? | ? | ? | ? | ? | ? | ? | ? |
|  |  | 6 | 64 | 64 | ? | ? | ? | ? | 160 | 160 | ? | ? | ? | ? | ? | ? | ? | ? | ? | ? | ? | ? | ? |
|  | tp | 1 | ? | ? | ? | 65 | ? | ? | ? | ? | ? | ? | ? | ? | ? | ? | ? | ? | ? | ? | ? | ? | ? |
| PP |  |  | ? | ? | ? | ? | ? | ? | ? | ? | ? | ? | ? | ? | ? | ? | ? | ? | ? | ? | ? | ? | ? |

**Supplementary file 1b. Length/width ratio of *Beretella* and *Saccorhytus*.**

| specimen number | L (in μm) | w (in μm) | L/H ratio  (elongation index) |
| --- | --- | --- | --- |
| *Beretella spinosa* gen. et sp. nov. from Cambrian Stage 2 Yanjiahe Formation in Yichang, China | | | |
| CUBar138-12 | 1960 | 1225 | 1.60 |
| CUBar121-8 | 2616 | 1972 | 1.33 |
| CUBar75-45 | 1170 | 975 | 1.20 |
| CUBar136-9 | 1411 | 1296 | 1.09 |
| CUBar171-5 | 1470 | 1159 | 1.27 |
| *Saccorhytus coronarius* from Cambrian Fortunian Kuanchuanpu Formation in Xixiang, China | | | |
| XX25-62 | 558 | 513 | 1.09 |
| XX27-168 | 870 | 560 | 1.55 |
| XX34-298 | 1079 | 752 | 1.43 |
| XX42-83 | 930 | 723 | 1.29 |
| XX44-301 | 1064 | 823 | 1.29 |
| XX45-1 | 1071 | 1010 | 1.06 |
| XX45-20 | 765 | 754 | 1.02 |
| XX45-56 | 1157 | 862 | 1.34 |
| XX48-64 | 986 | 779 | 1.27 |
| XX49-183 | 798 | 672 | 1.19 |
| XX50-360 | 549 | 426 | 1.29 |
| XX51-326 | 969 | 730 | 1.33 |
| XX55-43 | 1085 | 856 | 1.27 |
| XX56-493 | 1046 | 783 | 1.33 |
| XX58-331 | 1108 | 897 | 1.24 |
| XX61-27 | 1315 | 1003 | 1.31 |
| XX65-53 | 1090 | 871 | 1.25 |
| XX65-269 | 1400 | 1150 | 1.22 |
| XX71-118 | 941 | 712 | 1.32 |
| XX72-260 | 1510 | 1070 | 1.41 |
| XX76-42 | 1100 | 983 | 1.12 |
| XX79-17 | 1158 | 860 | 1.35 |
| XX84-373 | 1314 | 848 | 1.55 |
| XX114-139 | 1260 | 840 | 1.50 |

**Supplementary file 1c. Similarities and differences between *Beretella* and *Saccorhytus*.** AP, antero-posterior; DV, dorso-ventral side; LR, left-right.

|  | **overall shape** | **body polarities** | | | **body size**  **(in mm)** | **anus** | **spiny sclerites** | | | | **body rigidity** |
| --- | --- | --- | --- | --- | --- | --- | --- | --- | --- | --- | --- |
|  |  | AP | DV | LR |  |  | shape | number of large sclerites | arrangement | external ornament |  |
| *Saccorhytus* | sac-like | weak | strong | weak | 0.5-1.2, meiofaunal | absence | circular | five/six pairs | bilateral | radial wrinkles | soft |
| *Beretella* | beret-like | strong | strong | strong | 1.0-2.9, meiofaunal | absence | elliptical | two pairs | bilateral | reticulation (polygons) | possible partly sclerotized |
| Scalidophora | vermiform with introvert | strong | -weak | weak | >1.4, meiofaunal to macrofaunal | presence | elliptical to circular | up to 20 pairs | radial or bilateral | reticulation (polygons) | flexible cuticle (except sclerites) |

**Supplementary file 1d. Ancestral character state reconstructions for the topology where Cycloneuralia is monophyly.** Values of ancestral character state reconstructions. 0 = absence of character, 1 = presence of character, PP = posterior probability. TGE, total-group Ecdysozoa; CGE, crown-group Ecdysozoa, SA, Saccorhytida.

| Character | TGE(PP) | SA(PP) | CGE(PP) | Presence in *Beretella* |
| --- | --- | --- | --- | --- |
| Through gut | 0 = 0.035817 | 0 = 0.480277 | 0 = 0.000288 | uncertain |
|  | **1 = 0.964183** | 1 = 0.519723 | **1 = 0.999712** |  |
| Ventral mouth | 0 = 0.138924 | 0 = 0.081620 | **0 = 0.989498** | inferred |
|  | **1 = 0.861076** | **1 = 0.918380** | 1 = 0.010502 |  |
| Introvert | **0 = 0.999942** | **0 = 0.999976** | **0 = 0.999925** | no |
|  | 1 = 0.000058 | 1 = 0.000024 | 1 = 0.000075 |  |

**Supplementary file 1e. Ancestral character state reconstructions for the topology where Cycloneuralia is paraphyletic.** Values of ancestral character state reconstructions. 0 = absence of character, 1 = presence of character, PP = posterior probability. TGE, total-group Ecdysozoa; CGE, crown-group Ecdysozoa, SA, Saccorhytida.

| Character | TGE(PP) | SA(PP) | CGE(PP) | Presence in *Beretella*? |
| --- | --- | --- | --- | --- |
| Through gut | 0 = 0.037441 | 0 = 0.481858 | 0 = 0.000251 | uncertain |
|  | **1 = 0.963104** | 1 = 0.518142 | **1 = 0.999749** |  |
| Ventral mouth | 0 = 0.140461 | 0 = 0.082373 | **0 = 0.991707** | inferred |
|  | **1 = 0.859539** | **1 = 0.917627** | 1 = 0.008293 |  |
| Introvert | **0 = 0.999846** | **0 = 0.999972** | **0 = 0.996463** | no |
|  | 1 = 0.000154 | 1 = 0.000028 | 1 = 0.003537 |  |
